# What evidence exists regarding the effects of photovoltaic panels on biodiversity? A critical systematic map protocol

**DOI:** 10.1186/s13750-022-00291-x

**Published:** 2022-11-29

**Authors:** Alix Lafitte, Romain Sordello, Véronique de Crespin de Billy, Jérémy Froidevaux, Philippe Gourdain, Christian Kerbiriou, Joseph Langridge, Geoffroy Marx, Bertrand Schatz, Chloé Thierry, Yorick Reyjol

**Affiliations:** 1 PatriNat (Office Français de la Biodiversité (OFB) – Centre National de la Recherche Scientifique (CNRS) – Muséum National d’Histoire Naturelle (MNHN)), 75005 Paris, France; 2 OFB (Office Français de la Biodiversité), 94300 Vincennes, France; 3 CESCO UMR 7204, Muséum National d’Histoire Naturelle (MNHN), 75005 Paris, France; 4 University of Stirling, Biological and Environmental Sciences, Stirling, FK9 4LA Scotland, UK; 5 FRB–Cesab (Fondation pour la Recherche sur la Biodiversité – Centre de Synthèse et d’Analyse sur la Biodiversité), 75005 Paris, France; 6 LPO (Ligue pour la Protection des Oiseaux), 17300 Rochefort, France; 7 CEFE (Centre d’Ecologie Fonctionnelle & Evolutive), Université de Montpellier, CNRS (Centre National pour la Recherche Scientifique), EPHE (Ecole Pratique des Hautes Etudes), IRD (Institut de Recherche pour le Développement), 34090 Montpellier, France

**Keywords:** Conservation, Ecological transition, Floating solar, Floatovoltaics, Green infrastructure, Solar panels, Utility-scale solar energy, USSE, Wildlife

## Abstract

**Background:**

Climate change and the current phase-out of fossil fuel-fired power generation are currently expanding the market of renewable energy and more especially photovoltaic (PV) panels. Contrary to other types of renewable energies, such as wind and hydroelectricity, evidence on the effects of PV panels on biodiversity has been building up only fairly recently. PV panels have been linked to substantial impacts on species and ecosystems, the first and most obvious one being the degradation of natural habitats but they may also lead to mortality of individuals and displacements of populations. Hence, we propose a systematic map aiming to draw a comprehensive panorama of the available knowledge on the effects of photovoltaic and solar thermal (PVST) installations, whatever their scales (i.e. cells, panels, arrays, utility-scale facilities), on terrestrial and semi-aquatic species and natural/semi-natural habitats and ecosystems. This work aims at providing decision-makers with a better understanding of the effects of PVST installations and, therefore, help them further protect biodiversity while also mitigating anthropogenic climate change.

**Methods:**

We will follow the collaboration for environmental evidence guidelines and search for relevant peer-reviewed and grey literature in English or French. The search string will combine population (all wild terrestrial and semi-aquatic species—e.g. animals, plants, fungi, microorganisms—as well as natural/semi-natural terrestrial habitats and ecosystems) and exposure/intervention (all technologies of PVST panels at all scales of installations and therefore excluding concentrated solar power) terms. A pre-built test list of relevant articles will be used to assess the comprehensiveness of the search string. Extracted citations will be screened at title and full-text stages thanks to pre-defined inclusion/exclusion criteria. Accepted citations will then be split into studies and observations, from which relevant metadata (e.g. taxon, exposure/intervention, outcome) will be extracted and their internal validity assessed through a critical appraisal. The database will be accessible alongside a map report which will draw a landscape of eligible studies. By describing studied populations, exposures/interventions, outcomes and internal study validity results, the report will identify potential knowledge clusters and gaps regarding the effects of PVST installations on biodiversity and ecosystems.

**Supplementary Information:**

The online version contains supplementary material available at 10.1186/s13750-022-00291-x.

## Background

The Earth is warming at an alarming rate due to rising concentrations of greenhouse gases produced predominantly by fossil fuel combustion [1]. In an attempt to mitigate anthropogenic climate change, renewable energy technologies are being scaled up, particularly through solar photovoltaic power which accounted for approximately 60% of worldwide renewable electricity capacity additions in 2021 [2]. Solar power seems to be one of the most promising sources of renewable energy to the extent that, in the IEA (International Energy Agency) roadmap to achieve a net zero CO_2_ future by 2050, solar power will have seen its capacity increased 20-fold between 2021 and 2050, thus representing the largest source of energy with one fifth of global supply [3].

The idea of producing electricity with solar energy is not new and date back to Becquerel’s first discovery of the photovoltaic effect in 1839 [4]. Nowadays, several technologies have been developed to make the most of this vast and endless pool of solar energy [5]. At the forefront, solar photovoltaic (PV) panels are made of semiconductors that convert sunlight into electricity. PV panels are manufactured from different materials, monocrystalline (and also polycrystalline) silicon being the most common one but numerous heavy metals (e.g. copper, cadmium, lead, silver) may also be used [5]. PV panels are currently deployed in various configurations: on top of roofs, on ground-mounted utility-scale facilities—often called Utility-Scale Solar Energy (USSE) facilities—or even on water so called floatovoltaics or floating PV/solar—e.g. on oceans, lakes, reservoirs, canals [6–8]. In addition, Solar Thermal (ST) panels have also been developed as heating systems and can be installed on rooftops or as utility-scale facilities [5]. While PV panels are flat plate solar collectors, ST panels often consists of tube solar collectors filled by a heat-transfer fluid which convert sunlight into thermal energy. Concentrated Solar Power (CSP) comprises mirrors focusing sunrays usually on a central tower or on a tube filled by heat-transfer fluids to produce vapour and electricity through a steam turbine [5]. Now, while all these technologies bring the hope of cutting global greenhouse gas emissions, they may also contribute to current biodiversity erosion, which, intertwined with climate change, is one of the most pressing issues of the Anthropocene [9,10].

**Fig. 1:**
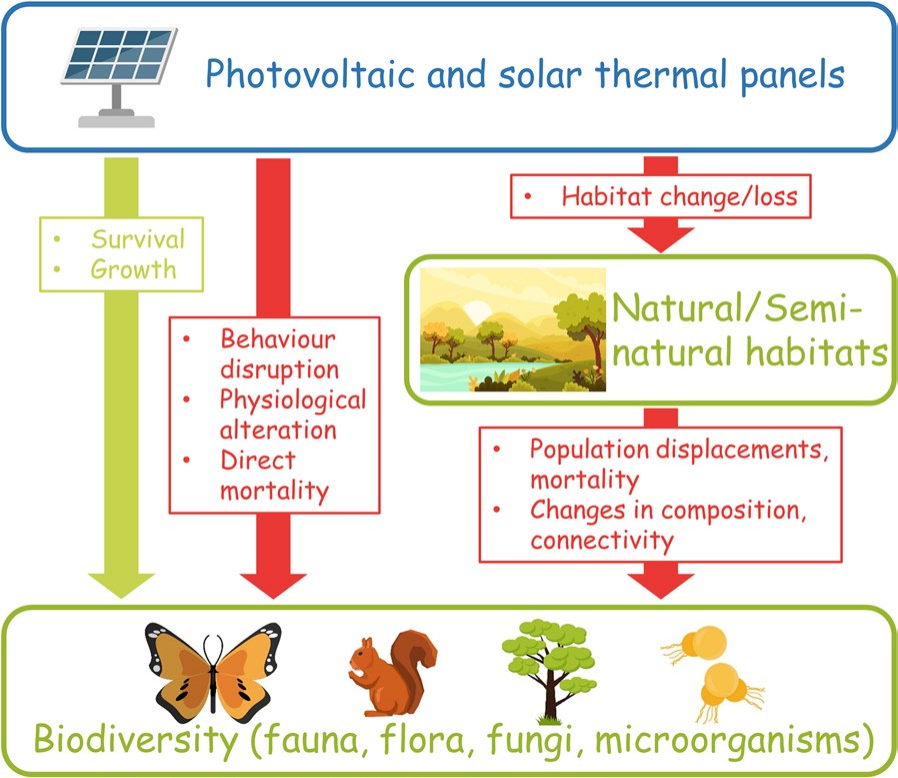
Conceptual model of photovoltaic and solar thermal panels potential effects on natural/semi-natural habitats and biodiversity. Green arrows represent potential positive outcomes and red arrows negative ones. Images designed by Freepik

Indeed, electricity generation can prove harmful to wildlife and ecosystems. For instance, thermal and nuclear power plants cause substantial avian fatalities notably due to extraction of primary resources, collisions with infrastructures and production of toxic wastes [11]. In addition, the combustion of fossil fuels like coal, oil and gas produces large amounts of fine particles and greenhouse gases, the latter being responsible for climate change, one of the main drivers of biodiversity erosion [9,12]. Renewable energies also have the potential to threaten species and ecosystems and while habitat change represents the main driver [13], other impacts on biodiversity have been reported as well: wind farms, like thermal power plants, may lead to bird and bat fatalities [14] whereas hydroelectricity impede fish migration routes and disrupt riparian communities [15]. As for solar energy and more especially PV installations, while evidence has been building up only fairly recently due to its relatively new entry into the market of energy production, they have already been linked to a wide range of negative impacts on species (Fig. 1): from mortality [16, 17], disruption of plant growth [18, 19] and animal behaviour [20–22], to alteration of population composition and diversity [23–25]. For instance, Horváth et al*.* [26] have shown that the strongly polarized light reflected by PV panels had the potential to lure aquatic insects, which then attempt to lay their eggs on these highly unsuitable surfaces. PV panels could thus become ecological traps reducing fitness and causing wide population declines. Graham et al*.* [27] investigated plant and pollinator populations under normal sunny conditions and under shade from PV panels and found a delayed plant phenology and bloom timing as well as a reduced pollinator abundance and richness under PV panels. Graham et al*.* [27] linked these ecosystem alterations to variations of microclimatic parameters associated with PV panels—e.g. soil temperature, soil moisture or solar radiation. Other studies indeed demonstrated higher humidity levels and warmer night-time temperatures around utility-scale PV facilities, so called photovoltaic heat island effect [28, 29]. In contrast with previous examples, other studies set in arid ecosystems have shown that these modified microclimatic conditions could prove beneficial to some plant communities. Liu et al*.* [30] for example, showed that solar PV facilities could promote plant biomass, coverage and richness therefore improving the progress and quality of vegetation recovery.

In addition, many other potential adverse effects of PV installations have been hypothesised in various technical reports—from French operational actors such as the ADEME (French Agency for Ecological Transition) [31], the FNE (France Nature Environment) [32] or the LPO (French Bird Conservation Association, unpublished) to international instances like the IUCN (International Union for Conservation of Nature) [33]—and reviews [8, 34, 35] but little empirical evidence is usually provided by authors [36]. For instance, PV installations have been hypothesised to promote exotic species invasions because of soil disturbances, lead to habitat fragmentation due to fences surrounding solar power infrastructures and generate soil erosion and loss due to dust generation and modified runoffs from PV panels [8, 35, 37] as well as contribute to chemical and noise pollution [34]. However, in most reviews, authors discussed the processes likely to occur at PV installations based on their own assumptions or extrapolating from the impacts observed on other man-made infrastructures built in similar environments. As for ST panels, we were not able to read any primary research articles or reviews on the matter. To our knowledge, a clear systematic map synthesizing all available evidence on the effects of photovoltaic—either USSE, floatovoltaics or on roofs—and solar thermal (PVST) installations on biodiversity is lacking. Review authors also rarely provided their literature search strategies, nor an easily accessible database of the literature they collated, nor did they attempt to assess the internal validity of the studies they discussed. Additionally, to protect wildlife while still mitigating anthropogenic climate change, we believe that there exists a need to better inform decision-makers as well as to guide future research. Hence, we propose to conduct a critical systematic map aiming at collating all available evidence regarding the effects of PVST installations on terrestrial and semi-aquatic biodiversity whatever their scales (i.e. cells, panels, arrays, utility-scale facilities). A systematic map report will be produced alongside an open-access database which will provide relevant metadata for all included studies.

### Stakeholder engagement

The FRB (French Foundation for Research on Biodiversity) launched a call to conduct systematic maps in order to study anthropogenic impacts on terrestrial biodiversity. Our team applied to this call and proposed a map aimed at building a comprehensive panorama of the available evidence on the effects of PVST installations, whatever their scales (i.e. cells, panels, arrays, utility-scale facilities), on biodiversity, which was later accepted by the FRB. The latter is part of the steering committee which will provide methodological expertise and follow the progress of this map. The FRB board is made up of 20 directors from eight French public research institutes as well as the corporate group LVMH, the Ineris (French National Institute for Industrial Environment and Risks), the University of Montpellier and the OFB (French Office for Biodiversity). The FRB’s principal mission is to support and conduct research through scientific cooperation as well as to increase and then transfer knowledge on biodiversity-related issues. Additionally, we identified a group of specialists on the matter of the ecological effects of PVST installations on biodiversity. Working at the French National Museum of Natural History (MNHN), the OFB, the LPO, the French research Centre of Evolutionary and Functional Ecology (CEFE) and the University of Stirling, these experts helped us better identify the contour of our study, build the search string, define the eligibility inclusion/exclusion criteria as well as assess the validity of metadata coding information.

## Objective of the map

The objective of this map is to draw an exhaustive panorama of the available knowledge on the effects of PVST installations, whatever their scales (i.e. cells, panels, arrays, utility-scale facilities), on biodiversity by building a comprehensive database and by highlighting any potential knowledge gaps or clusters where more focused systematic reviews could be contemplated.

### Primary question

The primary question is: what evidence exists regarding the effects of PVST installations, whatever their scales (i.e. cells, panels, arrays, utility-scale facilities), on wild terrestrial and semi-aquatic species?

### Component of the primary question

The above primary question has the following Population–Exposure/Intervention–Comparator–Outcome (PE/ICO) elements:

- Populations: All wild terrestrial and semi-aquatic species found globally (i.e. animals, plants, fungi, microorganisms living fully or partially in natural/semi-natural terrestrial habitats and ecosystems) excluding humans, domesticated and cultivated species as well as strictly aquatic ones (e.g. algae, fishes).
- Exposures/Interventions: All technologies of PVST panels whatever their configurations (i.e. on roofs, ground, or water) will be retained. All scales of PVST installations will be included whether it be cells, panels, arrays, or wider utility-scale facilities. Real and simulated experimental PVST panels will both be kept. The whole lifecycle of utility-scale PVST facilities (i.e. construction, operation and dismantlement phases) will be considered whereas the lifecycle of PVST panels will be excluded (i.e. material extraction, manufacturing and recycling phases). Even though Concentrated Solar Power (CSP) technologies rely on solar energy, they will not be included in this study.
- Comparators: Studies comparing a population exposed to a PVST installation and a population left unexposed and/or studies comparing a population before and after the construction of a PVST installation will be considered (Before-After temporal comparator and/or Control-Exposure/Intervention spatial comparator—e.g. BACE/I, BAE/I, CE/I). Studies comparing different types of PVST installations (e.g. technologies) will be included. On the contrary, studies without any comparator will not be retained.
- Outcomes: All outcomes related to the studied population will be considered (e.g. mortality, diversity, abundance, growth, distribution, physiology, reproduction, mobility, morphology, behaviour, habitat alteration, habitat connectivity, etc.). All abiotic parameters related to the studied natural/semi-natural habitat or ecosystem will be excluded.

### Secondary questions

The secondary questions are: what are the most studied species, habitats and ecosystems? What are the characteristics of the studied PVST installations (i.e. panel technology, size, fencing, type of management)? Which types of outcomes are more usually investigated? In which country and climatic zones studies have been carried out? What level of reliability can be granted to the studies that will be included in this systematic map?

## Methods

This systematic map will follow the Collaboration for Environmental Evidence Guidelines for Evidence Synthesis in Environmental Management [38] and it will comply with ROSES reporting standards [39] (see Additional file 1).

### Search for literature

#### Languages

Searches will be performed using exclusively English terms. Only studies published in English or French will be retained in this systematic map. We acknowledge that only including articles in these two languages constitutes a potential bias to our systematic map but this could not be avoided based the linguistic competences of the review team. The list of search terms is presented in the section below (see “Search string” section).

#### Search string

A scoping exercise was carried out on the Web of Science Core Collection (WOSCC) database in order to build the search string. Several combinations of search terms describing the population and exposure/intervention were trialled in order to reach the best comprehensiveness and specificity. The following search string (Web of Science format) is the result of this iterative process:

TS = ((photovoltaic$ OR "solar panel$" OR “solar array$” OR “solar development$” OR “solar power” OR “solar park$” OR “solar installation$” OR “solar facilit*” OR “solar plant$” OR “utility-scale solar energ*” OR “utility scale solar energ*” OR biosolar OR “float* solar” OR floatovoltaic$) AND (biodiversity OR ecolog* OR ecosystem$ OR wildlife OR “natural habitat$” OR species OR flora OR vegetation$ OR animal$ OR fauna OR vertebrate$ OR mammal$ OR bird$ OR reptile$ OR amphibian$ OR invertebrate$ OR arthropod$ OR insect$ OR arachnid$ OR crustacean$ OR mollus* OR microbi* OR bacteri* OR microorganism$ OR fung*)).

#### Comprehensiveness of the search

A test list of 26 relevant primary research articles was established (see Additional file 2) in order to assess the comprehensiveness of the literature search. These articles were identified by the review team, thanks to experts or through previous syntheses on PV installations and biodiversity carried out by French operational actors such as the ADEME [31], the LPO (unpublished) as well as the IUCN report [33].

#### Online publication databases

We conducted the search on four multidisciplinary databases: WOSCC, Biological Abstracts (BA), Zoological Records (ZR) (all from Clarivate Analytics) and Scopus (Elsevier)—using the access rights respectively provided by the MNHN and the CNRS (French National Centre for Scientific Research). All databases were selected for their relevance in the matter of ecological studies and for easy search reproducibility and accessibility. The WOSCC search included the following citation indexes: Science Citation Index Expanded (SCI–EXPANDED, 1956–present), Social Sciences Citation Index (SSCI, 1975–present), Arts & Humanities Citation Index (A&HCI, 1975–present), Conference Proceedings Citation Index–Science (CPCI–S, 1990–present), Conference Proceedings Citation Index–Social Science & Humanities (CPCI–SSH, 1990–present), Book Citation Index–Science (BKCI–S, 2005–present), Book Citation Index–Social Sciences & Humanities (BKCI–SSH, 2005–present), Emerging Sources Citation Index (ESCI, 2017–present), Current Chemical Reactions (CCR–EXPANDED, 1985–present) and Index Chemicus (IC, 1993–present). As for BA, ZR and Scopus, we had access to all indexed databases (respectively 1969–present, 1864–present and 1788–present). We adapted the WOSCC abovementioned search string to match the Scopus format for literature search (see Additional file 3). Among the 26 articles of the test list, 96% (25/26) are indexed in WOSCC and 96% (25/26) in Scopus (see Additional file 2), indicating a high degree of relevance of these two databases for our literature search. BA has an indexation level of 72% (18/26). On these three databases, one article from Bousselot et al*.* [40] was consistently not indexed. We checked its indexation in ZR but it was not present in this database either. On these four databases, our search string retrieves 11,053 citations of which 3,797 citations are found in WOSCC, 1,012 in BA, 102 in ZR, and 6,130 in Scopus. Among the 26 articles of the test list, our search string retrieves 100% (25/25) of articles indexed in WOSCC, 100% (25/25) in Scopus and 100% (18/18) in BA (see Additional file 2). Details on search hits from each selected database can be found in Additional file 3.

#### Internet searches

Additional searches will be performed using Google Scholar. The search string will be simplified and divided in four to fit the search facilities of this search engine—limited Boolean operators and a maximum of 256 characters [41] (see search strings in Additional file 3). Searches will be performed on titles exclusively. The first 250 hits of each search string will be retained in order to reach an addition of 1000 citations to the literature search. Results will be extracted using Publish or Perish (v 8.2.3944, downloaded on 07 June 2022) [42].

#### Specialist sources

We will search for relevant citations on the following additional specialist sites (English or French):

- IEA (International Energy Agency): https://www.iea.org/
- IRENA (International Renewable Energy Agency): https://www.irena.org/
- United States Office of Energy Efficiency & Renewable Energy: https://www.energy.gov/eere/office-energy-efficiency-renewable-energy
- OFATE (French and German Agency for Ecological Transition): https://energie-fr-de.eu/fr/accueil.html
- ADEME (French Agency for Ecological Transition): https://www.ademe.fr/

#### Supplementary searches

A call for literature will be conducted through a professional network to find non-peer reviewed literature in English and/or French—i.e. technical reports, M.Sc. thesis or Ph.D. thesis. Possible relevant citations identified by the review team throughout the process of making this map but not retrieved by the literature search may also be added. Due to time constraints, no forward or backward citation chasing—exploration of references from the literature collated in our final systematic map corpus—will be carried out.

### Article screening and study eligibility criteria

#### Screening process

After duplicate removal, citations will be first screened for eligibility on titles and then directly on full-texts. Screening will be performed by at least two reviewers whose decision consistency will be assessed *a priori* by computing the Randolph’s Kappa coefficient [43] on a random sample of 10% of references from our corpus. This proportion results from a compromise between high volumes of citations and time constraints and has usually been chosen in recent systematic maps and reviews—albeit the best and optimal practice would be, for each citation, to be screened once by each reviewer [44]. This process will be repeated until reaching a threshold value of 0.7 which we consider to be an acceptable level of agreement between reviewers. Before beginning the screening process independently, reviewers will meet to discuss and resolve all remaining disagreements. Eligibility criteria will then be redefined if necessary. At each stage of the screening process, special care will be taken to ensure no reviewer would screen their own articles.

#### Eligibility criteria

At the title screening stage, the eligibility of citations will be assessed on Population–Exposure/Intervention–Outcome criteria (Table 1). Strictly aquatic species will be excluded based on the demands of the stakeholders who commissioned this systematic map. However, as floating PVST panels may also impact aerial, terrestrial or semi-aquatic species such as birds, insects or amphibians, they will be included in our Exposure/Intervention criterion. We acknowledge that CSP may also be a substantial threat for biodiversity and the evidence regarding their impact should be summarised as well [8]. Nevertheless, as this technology relies on mirrors to collect solar energy and not on panels, we considered that both exposures were too different and therefore excluded CSP. Regarding natural/semi-natural habitats and ecosystems, while included in our population criterion, we will only focus on biotic outcomes resulting from PVST installations (e.g. lost area for wildlife) but we will not take into account citations strictly dealing with modifications of abiotic parameters (e.g. humidity, temperature, radiation). Citations elucidating the indirect effects of utility-scale PVST facilities (e.g. fencing, road, power line, evaporation pond) will be considered only if they are studied in the context of utility-scale PVST facilities and not extrapolated from other types of infrastructures. At full-text screening, complete Population–Exposure/Intervention–Comparator–Outcome criteria will be used as well as additional language, document type and content criteria (Table 2). We will consider all different contents being primary research, reviews, meta-analyses or modelling studies. Reviews and meta-analyses will be separated from the final corpus and their metadata coded in another additional file which will be appended to the final systematic map report. Conference objects (e.g. meeting abstracts, slides, posters) will be excluded because of their relatively low content in useful data and information. The list of excluded citations at the full-text stage will be provided alongside reasons for exclusion in an additional file.

**Table 1:** List of eligibility criteria at title screening

Include	Exclude
Populations	Populations
All wild terrestrial and semi-aquatic species found globally (i.e. animals, plants, fungi, microorganisms living fully or partially in natural/semi-natural terrestrial habitats and ecosystems)	Humans
	Domesticated or cultivated species
All natural/semi-natural habitats and ecosystems	Strictly aquatic or marine species (microalgae, fishes)
Exposures/interventions	Exposures/interventions
All technologies of photovoltaic and solar thermal (PVST) panels (e.g. monocrystalline, CdTe) whatever their configurations (i.e. on roofs, ground, or water)	Studies only on Concentrated Solar Power (CSP)
Real or simulated PVST panels	The lifecycle of PVST panels (i.e. material extraction, production and recycling phases)
All scales of PVST installations whether it be cells, panels, arrays, or wider utility-scale facilities	
The whole lifecycle of utility-scale PVST facilities (i.e. construction, operation and dismantlement phases)	
Outcomes	Outcomes
All outcomes related to the studied population (e.g. mortality, diversity, abundance, growth, distribution, physiology, reproduction, mobility, morphology, behaviour, habitat alteration, habitat connectivity, etc.)	All abiotic parameters related to the studied natural/semi-natural habitat or ecosystem

**Table 2:** List of additional eligibility criteria used at full-text screening

Include	Exclude
Comparators	Comparators
Studies comparing a population exposed to a photovoltaic and solar thermal (PVST) installation and a population left unexposed and/or studies comparing a population before and after the construction of a PVST installation—Before-After temporal comparator and/or Control-Exposure/Intervention spatial comparator (e.g. BACE/I, BAE/I, CE/I)	Studies without any comparator
Studies comparing different types of PVST installations	
Languages	
Articles written in English or French	
Document types	Document types
Journal article, book chapter, technical report, Ph.D. or M.Sc. thesis	Conference objects (e.g. meeting abstracts, slides, posters)
Document contents	
Primary research articles, reviews, meta-analyses, modelling studies without experimental data	

Based on these eligibility criteria, we undertook the estimation of our systematic map final volume of accepted citations. First, we removed all duplicates from our extracted references (see “Online publication databases” section) and selected a random subset of 100 citations. Ten references were included at title screening, among which 8 had an available full-text. This low proportion of accepted citations allowed us to consider skipping abstract screening and directly screen full-texts. As full-texts are more informative than abstracts, this method will improve the accuracy and robustness of the whole citation selection process. As such, on the subset of citations, full-text screening was directly performed which resulted in only one accepted article (see Additional file 4). As our search strategy resulted in approximately 8000 citations after duplicate removal, we expect to screen 800 full-texts and accept around 80 articles in our systematic map final corpus.

### Study validity assessment

All primary research articles accepted after screening will be split into studies—one study referring to one experimental design—and each study will be submitted to an internal validity assessment. Carrying out this critical appraisal is warranted as we expect non-peer reviewed studies (i.e. grey literature) to be included in this systematic map. By assessing studies’ level of robustness and confidence, we hope this map will offer a complete and reliable description of the actual state of the literature (i.e. knowledge gaps and clusters) which will save time for eventual future systematic reviews.

A Critical Appraisal Tool (CAT) was developed by the review team and will allow the assessment of both experimental and observational primary research studies. We will use the criteria identified in the CEECAT [45]—i.e. Confounding factors, Post-exposure/intervention selection, Misclassified comparison, Performance, Detection, Outcome reporting and Outcome assessment risks of biases. As allowed in CEE guidelines [38] and Frampton et al. [44], we decided to add an additional exposure risk of biases criterion which will assess whether experiments have been carried out on simulated PVST panels—for example, plastic sheeting on wood panels [46]. Even though such studies are within the scope of our systematic map, we wanted to take into account the potential high levels of confounding factors and thus high risk of biases, notably regarding the differences of microclimatic conditions between simulated and real PVST panels. We chose to adapt CEECAT questions and decision trees to better match the context of this map as well as time and high volume constraints related to the exercise of systematic mapping. As our internal validity assessing questions can be answered by a binary Yes or No, we will only assign studies with a low or high risk of bias rating—as well as an unclear rating for studies with insufficiently accurate or unknown information [44] (see Table 3, Additional file 5). In the end, a study’s overall risk of biases will be classified as low if all questions have low risks of biases, unclear if at least one question have an unclear risk of biases and high if at least one question have a high risk of biases. As reviews and meta-analyses will have been separated in another additional file, they will not be submitted to any critical appraisal. Studies’ external validity will not be evaluated in this systematic map as we will only assess the validity of the general knowledge base on the effects of PVST installations on biodiversity and will not attempt to answer a precise systematic review question.

**Table 3:** Studies internal validity critical appraisal tool

Risk of biases	Question	Low	High	Unclear
Confounding factors	Are there potential confounding factors influencing the exposure/intervention or outcome? (e.g. different ecosystems between sites, additional uncontrolled exposures such as light, chemical or noise pollution)	No or seemingly no	Yes or seemingly yes	Unknown or unclear
Post-exposure/intervention selection	Are experimental sites/groups randomly and/or systematically selected and exchangeability can be assumed after the exposure/intervention?	Yes or seemingly yes	No or seemingly no	Unknown or unclear
Attrition	Were there any differences in missing data between experimental sites/groups during the study or the analysis?	No or seemingly no	Yes or seemingly yes	Unknown or unclear
Misclassified comparison (only observational)	Are exposure/intervention and comparator groups sufficiently well defined?	Yes or seemingly yes	No or seemingly no	Unknown or unclear
Performance (only experimental)	Was the exposure/intervention altered during the experiment and thus differed between experimental sites/groups?	No or seemingly no	Yes or seemingly yes	Unknown or unclear
Detection	Are they differences in how outcomes were measured between experimental groups/sites?	No or seemingly no	Yes or seemingly yes	Unknown or unclear
Outcome reporting	Are reported findings selectively disclosed?	No or seemingly no	Yes or seemingly yes	Unknown or unclear
Outcome assessment	Were assumptions for the applied statistical analyses violated? (e.g. normality, homoscedasticity)	No or seemingly no	Yes or seemingly yes	Unknown or unclear
Exposure	Are real (not simulated) photovoltaic or solar thermal panels used?	Yes or seemingly yes	No or seemingly no	Unknown or unclear

Adapted from the Collaboration for Environmental Evidence Critical Appraisal Tool (CEECAT) [45]

Our critical appraisal tool was pilot-tested on a subsample of 5 articles coming from the test list (accounting for 8 studies). During the mapping process, before beginning critical appraisal, a random subset of 5% of accepted articles will be assessed by two reviewers. Reviewers will then meet to discuss and resolve all of their potential disagreements. Then, all remaining studies will be independently critically appraised by one of the two reviewers. At the end of the validity assessment stage, one reviewer will cross-check 5% of articles critically appraised by the other reviewer. This will result in 15% of the final corpus being critically appraised by both reviewers. We will make sure that no reviewer will critically appraise their own articles. Each overall risk of biases will finally be appended to its study in the metadata coding form in order to allow a direct assessment of each study internal validity by readers.

### Data coding strategy

As one study (i.e. one experimental design) can investigate several different populations and/or outcomes, all primary research studies will be split into observations—each observation referring to one outcome and one species. All observations will have their metadata coded in a coding form according to a pre-identified list of relevant variables (see codebook in Additional file 6), which were pilot-tested and refined from a subsample of 5 articles coming from the test list (accounting for 17 observations). The key variables will include:

- Bibliographic information (article, study and observation unique identifiers, authors, title, year, journal, DOI, language, publication type, publication content)
- Review information (reviewer, study internal validity assessment results)
- Study design (location, climatic zone, experimental designs, etc.)
- Description of the population (species, taxonomic group)
- Description of the exposure/intervention (technology, size, context, etc.)
- Description of the type of outcomes related to mortality, diversity, abundance, growth, distribution, physiology, reproduction, mobility, morphology, behaviour, community, habitat alteration, habitat connectivity for example. Other outcomes categories might be identified and added when coding for metadata.

Climatic zones will be identified thanks to the Köppen–Geiger climate classification which will be displayed on a Google Earth layer [47]. Metadata coding will be conducted independently by two reviewers whose agreement will be discussed and resolved *a priori* on a random subset of 5% of articles. At the end of metadata extraction phase, one reviewer will cross-check 5% of articles extracted by the other reviewer. This will result in 15% of the final corpus being extracted by both reviewers.

### Study map and presentation

An open-access database of all included articles, studies internal validity assessment results and observations data will be appended to the systematic map report. A narrative synthesis will be conducted and descriptive statistics, figures and tables will be used to describe internal validity assessment results, study designs, types of exposure/intervention as well as studied populations and outcomes. Population–Exposure/Intervention, Population–Outcome and Exposure/Intervention–Outcome crossing matrices in the form of heat maps or tables will be produced in order to identify the knowledge clusters and gaps regarding the effects of PVST installations on biodiversity. These results will help identify if a systematic review could be contemplated as well as which possible areas of research should be further investigated in the future. Potential updates to this map will be discussed in the ‘Implication for research’ section of the systematic map report based on the volume of recent publications found in the final corpus.

## Supplementary Information

**Additional file 1**. ROSES form for systematic map protocol.

**Additional file 2**. Test list indexation and comprehensiveness.

**Additional file 3**. Search string building process and results.

**Additional file 4**. Screening pilot-testing.

**Additional file 5**. Critical appraisal sheet.

**Additional file 6**. Codebook of systematic map database.

## Data Availability

The datasets supporting the conclusions of this article are included within the article and its additional files.
