# Supplementary material for: What evidence exists regarding the effects of photovoltaic panels on biodiversity? A critical systematic map protocol
Source: Environ Evid. 2022 Nov 29;11:36. doi: 10.1186/s13750-022-00291-x (PMC11378789; doi:10.1186/s13750-022-00291-x)
Supplement: Supplementary file 3 — Additional file 3. Search string building process and results. [file 13750_2022_291_MOESM3_ESM.docx]

**Additional file 3. Search string building process and results**

# **Search strings and intermediary search results**

| Date | Literature database | Search string | Volume | Comprehensiveness | Comments |
| --- | --- | --- | --- | --- | --- |
| 17/06/2022 | WOSCC | TS = ((photovoltaic$ OR “solar panel$” OR “solar array$” OR “solar development$” OR “solar power” OR “solar park$” OR “solar installation$” OR “solar facilit*” OR “solar plant$” OR “utility-scale solar energ*” OR “utility scale solar energ*” OR biosolar OR “float* solar” OR floatovoltaic$) AND (biodiversity OR ecolog* OR ecosystem$ OR wildlife OR “natural habitat$” OR species OR flora OR vegetation$ OR animal$ OR fauna OR vertebrate$ OR mammal$ OR bird$ OR reptile$ OR amphibian$ OR invertebrate$ OR arthropod$ OR insect$ OR arachnid$ OR crustacean$ OR mollus* OR microbi* OR bacteri* OR microorganism$ OR fung*)) | 3,797 | 100 % (25/25 indexed) | NA |
| 17/06/2022 | Biological Abstracts | TS = ((photovoltaic$ OR “solar panel$” OR “solar array$” OR “solar development$” OR “solar power” OR “solar park$” OR “solar installation$” OR “solar facilit*” OR “solar plant$” OR “utility-scale solar energ*” OR “utility scale solar energ*” OR biosolar OR “float* solar” OR floatovoltaic$) AND (biodiversity OR ecolog* OR ecosystem$ OR wildlife OR “natural habitat$” OR species OR flora OR vegetation$ OR animal$ OR fauna OR vertebrate$ OR mammal$ OR bird$ OR reptile$ OR amphibian$ OR invertebrate$ OR arthropod$ OR insect$ OR arachnid$ OR crustacean$ OR mollus* OR microbi* OR bacteri* OR microorganism$ OR fung*)) | 1,012 | 100 % (18/18 indexed) | NA |
| 17/06/2022 | Zoological Records | TS = ((photovoltaic$ OR “solar panel$” OR “solar array$” OR “solar development$” OR “solar power” OR “solar park$” OR “solar installation$” OR “solar facilit*” OR “solar plant$” OR “utility-scale solar energ*” OR “utility scale solar energ*” OR biosolar OR “float* solar” OR floatovoltaic$) AND (biodiversity OR ecolog* OR ecosystem$ OR wildlife OR “natural habitat$” OR species OR flora OR vegetation$ OR animal$ OR fauna OR vertebrate$ OR mammal$ OR bird$ OR reptile$ OR amphibian$ OR invertebrate$ OR arthropod$ OR insect$ OR arachnid$ OR crustacean$ OR mollus* OR microbi* OR bacteri* OR microorganism$ OR fung*)) | 102 | NA | NA |
| 20/06/2022 | Scopus | TITLE-ABS-KEY((photovoltaic OR “solar panel” OR “solar array” OR “solar development” OR “solar power” OR “solar park” OR “solar installation” OR “solar facilit*” OR “solar plant” OR “utility-scale solar energ*” OR “utility scale solar energ*” OR biosolar OR “float* solar” OR floatovoltaic) AND (biodiversity OR ecolog* OR ecosystem OR wildlife OR “natural habitat” OR species OR flora OR vegetation OR animal OR fauna OR vertebrate OR mammal OR bird OR reptile OR amphibian OR invertebrate OR arthropod OR insect OR arachnid OR crustacean OR mollus* OR microbi* OR bacteri* OR microorganism OR fung*)) | 6,130 | 100 % (25/25 indexed) | Cut in 4 date spans  (Scopus limitations):  - 1956 to 2009 (962 citations)  - 2010 to 2015 (1,659 citations)  - 2016 to 2019 (1,851 citations)  - 2019 to 2022 (1,658 citations) |
| 28/06/2022 | Google scholar | biodiversity AND (photovoltaic OR solar OR floatovoltaic OR floatovoltaics) | 59 | NA | NA |
| 28/06/2022 | Google scholar | vegetation AND (photovoltaic OR solar OR floatovoltaic OR floatovoltaics) | 250 | NA | 250 first hits out of 348 |
| 28/06/2022 | Google scholar | (animal OR animals) AND (photovoltaic OR solar OR floatovoltaic OR floatovoltaics) | 250 | NA | 250 first hits out of 284 |
| 28/06/2022 | Google scholar | (microorganism OR microorganisms) AND (photovoltaic OR solar OR floatovoltaic OR floatovoltaics) | 118 | NA | NA |
| Total | | | 11,718 |  |  |

# **Identification of relevant search terms**

1. Exposure terms:

- Photovoltaic solar cells / photovoltaic solar panels
- Photovoltaic panels
- PV installations
- Solar photovoltaic (power stations)
- Photovoltaic solar farms / Photovoltaic solar facility / solar photovoltaic farms
- Solar arrays / Photovoltaic arrays
- Solar power plants
- Solar power
- Solar park
- Solar installation
- Solar energy
- Utility-scale photovoltaic solar energy facility / Utility-scale solar energy facility / Utility-scale solar photovoltaic power plant / Photovoltaic utility scale solar facility
- Solar energy development
- Solar energy technologies
- Floatovoltaic / floating photovoltaic / Floating solar /floating solar panels
- Solar plant
- Monocrystalline panels
- Solar thermal energy/ Solar thermal plants
- Biosolar roofs
- Solar thermal
- Solar park
- Solar development
- Solar photovoltaic
- aquavoltaic (aquaculture + photovoltaic)

1. Population terms:

- Birds/Avian
- Pollinators
- Arthropods
- Flowers
- Fauna/flora
- Habitat/ microhabitat
- microclimate
- soils
- ecological
- biotic community / microbial communities / microbial biocenosis
- microorganisms / biofilms / microbiomes /microbial ecology / bacteria

# **WOSCC Search string building process:**

**Initial search string from Nov-Dec 2021**

| Step | Search string | Hits | Comprehensiveness | Comments |
| --- | --- | --- | --- | --- |
| Initial search string | (("photovoltaic panel$" OR "photovoltaic cell$" OR "solar panel$") AND (biodiversity OR ecosystem$ "natural habitat$" OR species OR ecolog* OR plant$ OR vegetation$ OR animal$ OR vertebrate$ OR mammal$ OR bird$ OR reptile$ OR amphibian$ OR invertebrate$ OR insect$ OR arachnid$ OR centipede$)) | ZR/WOSCC/BA :  TI = 41  TS = 4072  Scopus:  TI = 53  TS = 9645 | NA | NA |

**Test list search string**

DO = (10.1088/1748-9326/11/7/074016 10.1007/s10841-019-00191-5 10.1016/j.vetpar.2012.04.016 10.46534/jliv.2017.04.03.009 10.1016/j.landurbplan.2013.11.017 10.1038/srep29235 10.1038/s41598-021-86756-4 10.1002/ep.12626 10.3390/plants9091125 10.1111/j.1523-1739.2010.01518.x 10.1016/j.jhydrol.2018.11.019 10.1371/journal.pone.0232034 10.1002/ldr.3408 10.3389/fmicb.2018.03043 10.1016/j.jenvman.2018.08.017 10.1007/s00267-017-0906-4 10.1007/s10841-016-9897-3 10.1002/ecs2.3089 10.1002/eap.2349 10.1111/1758-2229.12608 10.1111/1751-7915.13620 10.1016/j.renene.2018.08.106 10.1016/j.renene.2016.02.041 10.1371/journal.pone.0243296 10.1080/15659801.2015.1045791) OR TI = (“REVEGETATION IN SOLAR PHOTOVOLTAIC FARMS IN MEDITERRANEAN AREAS”)

=> On the 26, 25 are indexed on Web of Science Core Collection on 3 June 2022

**Building process**

| **Date** | **Step** | **Search string** | **Hits (TS)** | **Comprehensiveness** | **Comments** |
| --- | --- | --- | --- | --- | --- |
| **23/05/2022** | 1 | (((photovoltaic OR solar) AND (panel$ OR cell$ OR array$ OR park$ OR energ* OR installation$ OR development OR power$ OR farm$ OR facilit* OR plant$ OR "utility scale" OR "utility-scale" OR float*) OR biosolar OR floatovoltaic$ OR thermal) AND (biodiversity OR ecosystem$ OR "natural habitat$" OR microhabitat$ OR microclimate$ Or soil$ OR species OR ecolog* OR plant$ OR flor* OR vegetation$ OR flower$ OR animal$ OR faun* OR vertebrate$ OR mammal$ OR bird$ OR avi* OR reptile$ OR amphibian$ OR invertebrate$ OR insect$ OR arthropd$ OR pollinator$ OR arachnid$ OR centipede$ OR microbi* OR bacteri* OR microorganism$ OR biocenosis)) | WOS:  221,998 | NA | Add majority of identified search terms; ‘thermal’ misplaced |
|  | 2 | (((photovoltaic OR solar) NEAR/5 (panel$ OR cell$ OR array$ OR park$ OR energ* OR installation$ OR development OR power$ OR farm$ OR facilit* OR plant$ OR "utility scale" OR "utility-scale" OR float*) OR biosolar OR floatovoltaic$ OR thermal) AND (biodiversity OR ecosystem$ OR "natural habitat$" OR microhabitat$ OR microclimate$ Or soil$ OR species OR ecolog* OR plant$ OR flor* OR vegetation$ OR flower$ OR animal$ OR faun* OR vertebrate$ OR mammal$ OR bird$ OR avi* OR reptile$ OR amphibian$ OR invertebrate$ OR insect$ OR arthropd$ OR pollinator$ OR arachnid$ OR centipede$ OR microbi* OR bacteri* OR microorganism$ OR biocenosis)) | 16,476 | NA | NEAR, AND: not valid in WOS format |
|  | 3 | (((photovoltaic OR solar) NEAR/5 (panel$ OR cell$ OR array$ OR park$ OR energ* OR installation$ OR development OR power$ OR farm$ OR facilit* OR infrastructure$ OR plant$ OR "utility scale" OR "utility-scale" OR float* OR thermal) OR biosolar OR floatovoltaic$) AND (biodiversity OR ecosystem$ OR "natural habitat$" OR microhabitat$ OR microclimate$ Or soil$ OR species OR ecolog* OR plant$ OR flor* OR vegetation$ OR flower$ OR animal$ OR faun* OR vertebrate$ OR mammal$ OR bird$ OR avi* OR reptile$ OR amphibian$ OR invertebrate$ OR insect$ OR arthropd$ OR pollinator$ OR arachnid$ OR centipede$ OR microbi* OR bacteri* OR microorganism$ OR biocenosis)) | 5,641 | NA | Get rid of ‘thermal physiology’ of animals; NEAR, AND: not valid in WOS format |
| **24/05/2022** | 1 | (((photovoltaic OR solar) NEAR/15 (panel$ OR cell$ OR array$ OR park$ OR energ* OR installation$ OR development OR power$ OR farm$ OR facilit* OR infrastructure$ OR plant$ OR float* OR thermal) OR biosolar OR floatovoltaic$) AND (biodiversity OR ecosystem$ OR "natural habitat$" OR microhabitat$ OR microclimate$ Or soil$ OR species OR ecolog* OR plant$ OR flor* OR vegetation$ OR flower$ OR animal$ OR faun* OR vertebrate$ OR mammal$ OR bird$ OR avi* OR reptile$ OR amphibian$ OR invertebrate$ OR insect$ OR arthropd$ OR pollinator$ OR arachnid$ OR centipede$ OR microbi* OR bacteri* OR microorganism$ OR biocenosis)) | 39,720 | NA | NEAR, AND: not valid in WOS format |
|  | 2 | (((photovoltaic OR solar) NEAR/10 (panel$ OR cell$ OR array$ OR park$ OR energ* OR installation$ OR development OR power$ OR farm$ OR facilit* OR infrastructure$ OR plant$ OR float* OR thermal) OR biosolar OR floatovoltaic$) AND (biodiversity OR ecosystem$ OR "natural habitat$" OR microhabitat$ OR microclimate$ Or soil$ OR species OR ecolog* OR plant$ OR flor* OR vegetation$ OR flower$ OR animal$ OR faun* OR vertebrate$ OR mammal$ OR bird$ OR avi* OR reptile$ OR amphibian$ OR invertebrate$ OR insect$ OR arthropd$ OR pollinator$ OR arachnid$ OR centipede$ OR microbi* OR bacteri* OR microorganism$ OR biocenosis)) | 37,988 | NA | High level of solar radiation and plant (#1 NOT #2) ; NEAR, AND: not valid in WOS format |
|  | 3 | (((photovoltaic OR solar) NEAR/5 (panel$ OR cell$ OR array$ OR park$ OR energ* OR installation$ OR development OR power$ OR farm$ OR facilit* OR infrastructure$ OR plant$ OR float* OR thermal) OR biosolar OR floatovoltaic$) AND (biodiversity OR ecosystem$ OR "natural habitat$" OR microhabitat$ OR microclimate$ OR soil$ OR species OR ecolog* OR plant$ OR flor* OR vegetation$ OR flower$ OR animal$ OR faun* OR vertebrate$ OR mammal$ OR bird$ OR avi* OR reptile$ OR amphibian$ OR invertebrate$ OR insect$ OR arthropd$ OR pollinator$ OR arachnid$ OR centipede$ OR microbi* OR bacteri* OR microorganism$ OR biocenosis)) | 35,281 | NA | High level of solar radiation and plant (#2 NOT #3) ; NEAR, AND: not valid in WOS format |
|  | 4 | (((photovoltaic OR solar) AND (panel$ OR cell$ OR array$ OR park$ OR energ* OR installation$ OR development OR power$ OR farm$ OR facilit* OR infrastructure$ OR plant$ OR float* OR thermal) OR biosolar OR floatovoltaic$) AND (biodiversity OR ecosystem$ OR "natural habitat$" OR microhabitat$ OR microclimate$ OR soil$ OR species OR ecolog* OR plant$ OR flor* OR vegetation$ OR flower$ OR animal$ OR faun* OR vertebrate$ OR mammal$ OR bird$ OR avi* OR reptile$ OR amphibian$ OR invertebrate$ OR insect$ OR arthropd$ OR pollinator$ OR arachnid$ OR centipede$ OR microbi* OR bacteri* OR microorganism$ OR biocenosis)) | 56,306 | NA | NA |
|  | 5 | (("photovoltaic panel$" OR "photovoltaic cell$" OR “photovoltaic array$” OR “photovoltaic power” OR "solar panel$" OR “solar array$” OR “solar cell$” OR “solar photovoltaic” OR “photovoltaic solar” OR “solar development$” OR “solar park$” OR “solar installation$” OR “solar facilit*” OR “solar energ*” OR “solar power” OR “solar plant$” OR “solar thermal” OR biosolar OR “float* photovoltaic$” OR “float* solar” OR floatovoltaic$) AND (biodiversity OR ecosystem$ OR "natural habitat$" OR microhabitat$ OR microclimate$ OR soil$ OR species OR ecolog* OR plant$ OR flor* OR vegetation$ OR flower$ OR animal$ OR faun* OR vertebrate$ OR mammal$ OR bird$ OR avi* OR reptile$ OR amphibian$ OR invertebrate$ OR insect$ OR arthropd$ OR pollinator$ OR arachnid$ OR centipede$ OR microbi* OR bacteri* OR microorganism$ OR biocenosis)) | 27,184 | NA | Split exposure expressions ; Much better sensibility compared to separated search terms |
|  | 6 | ((photovoltaic OR "solar panel$" OR “solar array$” OR “solar cell$” OR “solar development$” OR “solar park$” OR “solar installation$” OR “solar facilit*” OR “solar energ*” OR “solar power” OR “solar plant$” OR “solar thermal” OR biosolar OR “float* solar” OR floatovoltaic$) AND (biodiversity OR ecosystem$ OR "natural habitat$" OR microhabitat$ OR microclimate$ OR soil$ OR species OR ecolog* OR plant$ OR flor* OR vegetation$ OR flower$ OR animal$ OR faun* OR vertebrate$ OR mammal$ OR bird$ OR avi* OR reptile$ OR amphibian$ OR invertebrate$ OR insect$ OR arthropd$ OR pollinator$ OR arachnid$ OR centipede$ OR microbi* OR bacteri* OR microorganism$ OR biocenosis)) | 31,433 | NA | Try to separate photovoltaic ; a lot of noise from soil and solar energy but sometimes PV referred as solar energy |
|  | 7 | ((photovoltaic OR "solar panel$" OR “solar array$” OR “solar cell$” OR “solar development$” OR “solar park$” OR “solar installation$” OR “solar facilit*” OR “solar power” OR “solar plant$” OR “solar thermal” OR biosolar OR “float* solar” OR floatovoltaic$) AND (biodiversity OR ecosystem$ OR "natural habitat$" OR microhabitat$ OR microclimate$ OR species OR ecolog* OR plant$ OR flor* OR vegetation$ OR flower$ OR animal$ OR faun* OR vertebrate$ OR mammal$ OR bird$ OR avi* OR reptile$ OR amphibian$ OR invertebrate$ OR insect$ OR arthropd$ OR pollinator$ OR arachnid$ OR centipede$ OR microbi* OR bacteri* OR microorganism$ OR biocenosis)) | 24,703 | NA | Without solar energy and soils ; some relevant hit but a lot of noise still |
| **02/06/2022** | 1 | (("photovoltaic panel$" OR "photovoltaic cell$" OR "solar panel$") AND (biodiversity OR ecosystem$ "natural habitat$" OR species OR ecolog* OR plant$ OR vegetation$ OR animal$ OR vertebrate$ OR mammal$ OR bird$ OR reptile$ OR amphibian$ OR invertebrate$ OR insect$ OR arachnid$ OR centipede$)) | ZR/WOSCC/BA :  TS = 4,327 | NA | NA |
|  | 2 | ((photovoltaic OR "solar panel$" OR “solar array$” OR “solar cell$” OR “solar development$” OR “solar park$” OR “solar installation$” OR “solar facilit*” OR “solar plant$” OR “solar thermal” OR biosolar OR “float* solar” OR floatovoltaic$) AND (biodiversity OR ecosystem$ OR "natural habitat$" OR microhabitat$ OR microclimate$ OR species OR ecolog* OR plant$ OR flor* OR vegetation$ OR flower$ OR animal$ OR faun* OR vertebrate$ OR mammal$ OR bird$ OR avi* OR reptile$ OR amphibian$ OR invertebrate$ OR insect$ OR arthropd$ OR pollinator$ OR arachnid$ OR centipede$ OR microbi* OR bacteri* OR microorganism$ OR biocenosis)) | ZR/WOSCC/BA :  41,195 | NA | Without solar power |
|  | 3 | (("photovoltaic panel$" OR "photovoltaic cell$" OR “photovoltaic array$” OR “photovoltaic power” OR "solar panel$" OR “solar array$” OR “solar cell$” OR “solar photovoltaic” OR “photovoltaic solar” OR “solar development$” OR “solar park$” OR “solar installation$” OR “solar facilit*” OR “solar plant$” OR “solar thermal” OR biosolar OR “float* photovoltaic$” OR “float* solar” OR floatovoltaic$) AND (biodiversity OR ecosystem$ OR "natural habitat$" OR microhabitat$ OR microclimate$ OR species OR ecolog* OR plant$ OR flor* OR vegetation$ OR flower$ OR animal$ OR faun* OR vertebrate$ OR mammal$ OR bird$ OR avi* OR reptile$ OR amphibian$ OR invertebrate$ OR insect$ OR arthropd$ OR pollinator$ OR arachnid$ OR centipede$ OR microbi* OR bacteri* OR microorganism$ OR biocenosis)) | ZR/WOSCC/BA :  36,078  WOSCC: 15,358 | NA | Expressions but without solar power |
|  | 4 | (("photovoltaic panel$" OR "photovoltaic cell$" OR “photovoltaic array$” OR "solar panel$" OR “solar array$” OR “solar cell$” OR “solar photovoltaic” OR “photovoltaic solar” OR “float* photovoltaic$” OR “float* solar” OR floatovoltaic$) AND (biodiversity OR ecosystem$ OR "natural habitat$" OR microhabitat$ OR microclimate$ OR species OR ecolog* OR plant$ OR flor* OR vegetation$ OR flower$ OR animal$ OR faun* OR vertebrate$ OR mammal$ OR bird$ OR avi* OR reptile$ OR amphibian$ OR invertebrate$ OR insect$ OR arthropd$ OR pollinator$ OR arachnid$ OR centipede$ OR microbi* OR bacteri* OR microorganism$ OR biocenosis)) | ZR/WOSCC/BA :  18,078  WOSCC: 9,765 | NA | Huge simplification of E terms; loss of many relevant hit (solar facility) |
|  | 5 | (("photovoltaic panel$" OR "photovoltaic cell$" OR “photovoltaic array$” OR "solar panel$" OR “solar array$” OR “solar cell$” OR “solar photovoltaic” OR “photovoltaic solar” OR “float* photovoltaic$” OR “float* solar” OR floatovoltaic$) AND (biodiversity OR ecosystem$ OR "natural habitat$" OR microhabitat$ OR microclimate$ OR species OR ecolog* OR plant$ OR vegetation$ OR animal$ OR vertebrate$ OR mammal$ OR bird$ OR avi* OR reptile$ OR amphibian$ OR invertebrate$ OR insect$ OR arthropd$ OR pollinator$ OR arachnid$ OR centipede$ OR microbi* OR bacteri* OR microorganism$)) | WOSCC: 8,879 | NA | Simplification of P terms but loss of many relevant hits |
|  | 6 | (("photovoltaic panel$" OR "photovoltaic cell$" OR “photovoltaic array$” OR "solar panel$" OR “solar array$” OR “solar cell$” OR “solar photovoltaic” OR “photovoltaic solar” OR “solar facilit*” OR “float* photovoltaic$” OR “float* solar” OR floatovoltaic$) AND (biodiversity OR ecosystem$ OR "natural habitat$" OR microhabitat$ OR microclimate$ OR species OR ecolog* OR plant$ OR flor* OR vegetation$ OR flower$ OR animal$ OR faun* OR vertebrate$ OR mammal$ OR bird$ OR avi* OR reptile$ OR amphibian$ OR invertebrate$ OR insect$ OR arthropd$ OR pollinator$ OR arachnid$ OR centipede$ OR microbi* OR bacteri* OR microorganism$ OR biocenosis)) | WOSCC: 9,821 | NA | Return of solar facility |
|  | 7 | (("photovoltaic panel$" OR "photovoltaic cell$" OR “photovoltaic array$” OR "solar panel$" OR “solar array$” OR “solar cell$” OR “solar photovoltaic” OR “photovoltaic solar” OR “solar facilit*” OR “float* photovoltaic$” OR “float* solar” OR floatovoltaic$) AND (biodiversity OR ecosystem$ OR "natural habitat$" OR microhabitat$ OR microclimate$ OR species OR ecolog* OR flor* OR vegetation$ OR flower$ OR animal$ OR faun* OR vertebrate$ OR mammal$ OR bird$ OR avi* OR reptile$ OR amphibian$ OR invertebrate$ OR insect$ OR arthropd$ OR pollinator$ OR arachnid$ OR centipede$ OR microbi* OR bacteri* OR microorganism$ OR biocenosis)) | WOSCC:  6,009 | NA | Without plants  (a lot of noise from plants) |
|  | 8 | (("photovoltaic panel$" OR "photovoltaic cell$" OR “photovoltaic array$” OR “photovoltaic power” OR "solar panel$" OR “solar array$” OR “solar cell$” OR “solar photovoltaic” OR “photovoltaic solar” OR “solar development$” OR “solar park$” OR “solar installation$” OR “solar facilit*” OR “solar plant$” OR “solar thermal” OR biosolar OR “float* photovoltaic$” OR “float* solar” OR floatovoltaic$) AND (biodiversity OR ecosystem$ OR "natural habitat$" OR microhabitat$ OR microclimate$ OR species OR ecolog* OR flor* OR vegetation$ OR flower$ OR animal$ OR faun* OR vertebrate$ OR mammal$ OR bird$ OR avi* OR reptile$ OR amphibian$ OR invertebrate$ OR insect$ OR arthropd$ OR pollinator$ OR arachnid$ OR centipede$ OR microbi* OR bacteri* OR microorganism$ OR biocenosis)) | WOSCC:  6,408 | NA | More complete E search terms  => same comprehensiveness and much more reduced than 7th step from 24/05 reduced # 24,703 |
| **03/06/2022** | 1 | (("photovoltaic panel$" OR "photovoltaic cell$" OR “photovoltaic array$” OR “photovoltaic power” OR "solar panel$" OR “solar array$” OR “solar cell$” OR “solar photovoltaic” OR “photovoltaic solar” OR “solar development$” OR “solar park$” OR “solar installation$” OR “solar facilit*” OR “solar plant$” OR “solar thermal” OR biosolar OR “float* photovoltaic$” OR “float* solar” OR floatovoltaic$) AND (biodiversity OR ecosystem$ OR "natural habitat$" OR microhabitat$ OR microclimate$ OR species OR ecolog* OR flor* OR vegetation$ OR flower$ OR animal$ OR faun* OR vertebrate$ OR mammal$ OR bird$ OR avi* OR reptile$ OR amphibian$ OR invertebrate$ OR insect$ OR arthropd$ OR pollinator$ OR arachnid$ OR centipede$ OR microbi* OR bacteri* OR microorganism$ OR biocenosis)) | WOSCC: 6,413 | WOSCC: 23/25 = 92 % | Same step 8 of 02/06 |
|  | 2 | (("photovoltaic panel$" OR "photovoltaic cell$" OR “photovoltaic array$” OR “photovoltaic power” OR "solar panel$" OR “solar array$” OR “solar cell$” OR “solar photovoltaic” OR “photovoltaic solar” OR “solar development$” OR “solar park$” OR “solar installation$” OR “solar facilit*” OR “solar plant$” OR biosolar OR “float* photovoltaic$” OR “float* solar” OR floatovoltaic$) AND (biodiversity OR ecosystem$ OR "natural habitat$" OR microhabitat$ OR microclimate$ OR species OR ecolog* OR flor* OR vegetation$ OR flower$ OR animal$ OR faun* OR vertebrate$ OR mammal$ OR bird$ OR avi* OR reptile$ OR amphibian$ OR invertebrate$ OR insect$ OR arthropd$ OR pollinator$ OR arachnid$ OR centipede$ OR microbi* OR bacteri* OR microorganism$ OR biocenosis)) | 6,182 | WOSCC: 23/25 = 92 % | Without ‘solar thermal’, bring noise |
|  | 3 | (("photovoltaic panel$" OR "photovoltaic cell$" OR “photovoltaic array$” OR “photovoltaic power” OR "solar panel$" OR “solar array$” OR “solar cell$” OR “solar photovoltaic” OR “photovoltaic solar” OR “solar development$” OR “solar park$” OR “solar installation$” OR “solar facilit*” OR “solar plant$” OR biosolar OR “float* photovoltaic$” OR “float* solar” OR floatovoltaic$) AND (biodiversity OR ecosystem$ OR "natural habitat$" OR microhabitat$ OR microclimate$ OR species OR ecolog* OR flor* OR vegetation$ OR animal$ OR faun* OR vertebrate$ OR mammal$ OR bird$ OR reptile$ OR amphibian$ OR invertebrate$ OR insect$ OR arthropd$ OR pollinator$ OR microbi* OR bacteri* OR microorganism$)) | 5,341 | WOSCC: 23/25 = 92 % | Refined P terms (equalized and homogeneous)  => avi* = aviation, avionics  => flower : dye sensitive PV panels  => Much less noise too |
|  | 4 | (("photovoltaic panel$" OR "photovoltaic cell$" OR “photovoltaic array$” OR “photovoltaic power” OR "solar panel$" OR “solar array$” OR “solar cell$” OR “solar photovoltaic” OR “photovoltaic solar” OR “solar development$” OR “solar park$” OR “solar installation$” OR “solar facilit*” OR “solar plant$” OR “solar energ*” OR biosolar OR “float* photovoltaic$” OR “float* solar” OR floatovoltaic$) AND (biodiversity OR ecosystem$ OR "natural habitat$" OR microhabitat$ OR microclimate$ OR species OR ecolog* OR flor* OR vegetation$ OR animal$ OR faun* OR vertebrate$ OR mammal$ OR bird$ OR reptile$ OR amphibian$ OR invertebrate$ OR insect$ OR arthropd$ OR pollinator$ OR microbi* OR bacteri* OR microorganism$)) | 8,750 | WOSCC: 25/25 = 100 % | ‘solar energy’  => lot of noise : solar energy plant  Solar energy but PV ?  => A LOT OF NOISE => reduce test list ? |
|  | 5 | (("photovoltaic panel$" OR "photovoltaic cell$" OR “photovoltaic array$” OR “photovoltaic power” OR "solar panel$" OR “solar array$” OR “solar cell$” OR “solar photovoltaic” OR “photovoltaic solar” OR “solar development$” OR “solar park$” OR “solar installation$” OR “solar facilit*” OR “solar plant$” OR “utility-scale solar energ*” OR biosolar OR “float* photovoltaic$” OR “float* solar” OR floatovoltaic$) AND (biodiversity OR ecosystem$ OR "natural habitat$" OR microhabitat$ OR microclimate$ OR species OR ecolog* OR flor* OR vegetation$ OR animal$ OR faun* OR vertebrate$ OR mammal$ OR bird$ OR reptile$ OR amphibian$ OR invertebrate$ OR insect$ OR arthropd$ OR pollinator$ OR microbi* OR bacteri* OR microorganism$)) | 5,357 | WOSCC: 25/25 = 100 % | Utility-scale solar energy to reduce noise  => huge noise reduction |
|  | 6 | (("photovoltaic panel$" OR "photovoltaic cell$" OR “photovoltaic array$” OR “photovoltaic power” OR "solar panel$" OR “solar array$” OR “solar cell$” OR “solar photovoltaic” OR “photovoltaic solar” OR “solar development$” OR “solar park$” OR “solar installation$” OR “solar facilit*” OR “solar plant$” OR “utility-scale solar energ*” OR “float* photovoltaic$” OR “float* solar” OR floatovoltaic$) AND (biodiversity OR ecosystem$ OR "natural habitat$" OR microhabitat$ OR microclimate$ OR species OR ecolog* OR flor* OR vegetation$ OR animal$ OR faun* OR vertebrate$ OR mammal$ OR bird$ OR reptile$ OR amphibian$ OR invertebrate$ OR insect$ OR arthropd$ OR pollinator$ OR microbi* OR bacteri* OR microorganism$)) | 5,339 | WOSCC: 25/25 = 100 % | Biosolar ?  => relevant references |
|  | 7 | (("photovoltaic panel$" OR "photovoltaic cell$" OR “photovoltaic array$” OR “photovoltaic power” OR "solar panel$" OR “solar array$” OR “solar cell$” OR “solar photovoltaic” OR “photovoltaic solar” OR “solar development$” OR “solar park$” OR “solar installation$” OR “solar facilit*” OR “solar plant$” OR “utility-scale solar energ*” OR biosolar OR “float* photovoltaic$” OR “float* solar” OR floatovoltaic$) AND (biodiversity OR ecolog* OR ecosystem$ OR species OR flor* OR vegetation$ OR animal$ OR faun* OR vertebrate$ OR mammal$ OR bird$ OR reptile$ OR amphibian$ OR invertebrate$ OR insect$ OR arthropd$ OR pollinator$ OR microbi* OR bacteri* OR microorganism$)) | 5,296 | WOSCC: 25/25 = 100 % | Rearrange P for homogeneity |
|  | 8 | (("photovoltaic panel$" OR "photovoltaic cell$" OR “photovoltaic array$” OR “photovoltaic power” OR "solar panel$" OR “solar array$” OR “solar cell$” OR “solar photovoltaic” OR “photovoltaic solar” OR “solar development$” OR “solar park$” OR “solar installation$” OR “solar facilit*” OR “solar plant$” OR “utility-scale solar energ*” OR biosolar OR “float* photovoltaic$” OR “float* solar” OR floatovoltaic$) AND (biodiversity OR ecolog* OR ecosystem$ OR *habitat$ OR species OR flor* OR vegetation$ OR animal$ OR faun* OR vertebrate$ OR mammal$ OR bird$ OR reptile$ OR amphibian$ OR invertebrate$ OR insect$ OR arthropd$ OR pollinator$ OR microbi* OR bacteri* OR microorganism$)) | 5,325 | WOSCC: 25/25 = 100 % | With habitat, bring light additional noise  But relevant from fragmentation, connectivity, bring  noise with solar cell |
|  | 9 | (("photovoltaic panel$" OR "photovoltaic cell$" OR “photovoltaic array$” OR “photovoltaic power” OR "solar panel$" OR “solar array$” OR “solar cell$” OR “solar photovoltaic” OR “photovoltaic solar” OR “solar development$” OR “solar park$” OR “solar installation$” OR “solar facilit*” OR “solar plant$” OR “utility-scale solar energ*” OR biosolar OR “float* photovoltaic$” OR “float* solar” OR floatovoltaic$) AND (biodiversity OR ecolog* OR ecosystem$ OR *habitat$ OR species OR flor* OR vegetation$ OR animal$ OR faun* OR vertebrate$ OR mammal$ OR bird$ OR reptile$ OR amphibian$ OR invertebrate$ OR insect$ OR arthropd$ OR pollinator$ OR microbi* OR bacteri* OR microorganism$ OR fung* OR mushroom$)) | 5,376 | WOSCC: 25/25 = 100 % | With fungi/mushroom, bring additional noise from agrivoltaic fungi crop but might be relevant for actualisation |
|  | 10 | (("photovoltaic panel$" OR "photovoltaic cell$" OR “photovoltaic array$” OR “photovoltaic power” OR "solar panel$" OR “solar array$” OR “solar photovoltaic” OR “photovoltaic solar” OR “solar development$” OR “solar park$” OR “solar installation$” OR “solar facilit*” OR “solar plant$” OR “utility-scale solar energ*” OR biosolar OR “float* photovoltaic$” OR “float* solar” OR floatovoltaic$) AND (biodiversity OR ecolog* OR ecosystem$ OR *habitat$ OR species OR flor* OR vegetation$ OR animal$ OR faun* OR vertebrate$ OR mammal$ OR bird$ OR reptile$ OR amphibian$ OR invertebrate$ OR insect$ OR arthropod$ OR pollinator$ OR microbi* OR bacteri* OR microorganism$ OR fung* OR mushroom$)) | 1,420  WOSCC/ZR/BA : 3,202 | WOSCC: 25/25 = 100 %  WOSCC/ZR/BA = 100 % | Without solar cells, bring lot of noise of physical properties of cells (ion, metals) |
|  | 11 | (("photovoltaic panel$" OR "photovoltaic cell$" OR “photovoltaic array$” OR “photovoltaic power” OR "solar panel$" OR “solar array$” OR “solar photovoltaic” OR “photovoltaic solar” OR “solar development$” OR “solar park$” OR “solar installation$” OR “solar facilit*” OR “solar plant$” OR “utility-scale solar energ*” OR biosolar OR “float* photovoltaic$” OR “float* solar” OR floatovoltaic$) AND (biodiversity OR ecolog* OR ecosystem$ OR *habitat$ OR species OR flor* OR plant$ OR vegetation$ OR animal$ OR faun* OR vertebrate$ OR mammal$ OR bird$ OR reptile$ OR amphibian$ OR invertebrate$ OR insect$ OR arthropod$ OR pollinator$ OR microbi* OR bacteri* OR microorganism$ OR fung* OR mushroom$)) | WOSCC: 7,368  WOSCC/ZR/BA : 25,329 | WOSCC: 100 %  WOSCC/ZR/BA : 100 % | Plants, bring way too much noise  Because take all citations on ‘power plants’ without any ecological matters |
|  | 12 | (("photovoltaic panel$" OR "photovoltaic cell$" OR "solar panel$") AND (biodiversity OR ecosystem$ "natural habitat$" OR species OR ecolog* OR plant$ OR vegetation$ OR animal$ OR vertebrate$ OR mammal$ OR bird$ OR reptile$ OR amphibian$ OR invertebrate$ OR insect$ OR arachnid$ OR centipede$)) | ZR/WOSCC/BA :  TS = 4,330 | ZR/WOSCC/BA :  20/25 = 80 % | Test of initial equation comprehensiveness  Test Step 12 NOT Step 10: 3,020 different hits = only noise from plants (first 100 screened) |
| **10/06/2022** | 1 | ((photovoltaic$ OR "solar panel$" OR “solar array$” OR “solar development$” OR “solar park$” OR “solar installation$” OR “solar facilit*” OR “solar plant$” OR “utility-scale solar energ*” OR “utility scale solar energ*” OR biosolar OR “float* solar” OR floatovoltaic$) AND (biodiversity OR ecolog* OR ecosystem$ OR *habitat$ OR species OR flor* OR vegetation$ OR animal$ OR faun* OR vertebrate$ OR mammal$ OR bird$ OR reptile$ OR amphibian$ OR invertebrate$ OR insect$ OR arthropod$ OR pollinator$ OR microbi* OR bacteri* OR microorganism$ OR fung* OR mushroom$)) | 3,724 | 23/25 =  92 % | With criteria from Geoffroy Marx:  - synthesised photovoltaic  Good idea because some relevant hit |
|  | 2 | ((photovoltaic$ OR "solar panel$" OR “solar array$” OR “solar development$” OR “solar power” OR “solar park$” OR “solar installation$” OR “solar facilit*” OR “solar plant$” OR “utility-scale solar energ*” OR “utility scale solar energ*” OR biosolar OR “float* solar” OR floatovoltaic$) AND (biodiversity OR ecolog* OR ecosystem$ OR *habitat$ OR species OR flor* OR vegetation$ OR animal$ OR faun* OR vertebrate$ OR mammal$ OR bird$ OR reptile$ OR amphibian$ OR invertebrate$ OR insect$ OR arthropod$ OR pollinator$ OR microbi* OR bacteri* OR microorganism$ OR fung* OR mushroom$)) | 4,078 | 25/25 =  100 % | With solar power in addition  Many relevant hits (CSP)  and “utility scale” too |
|  | 3 | (("photovoltaic panel$" OR "photovoltaic cell$" OR “photovoltaic array$” OR “photovoltaic power” OR "solar panel$" OR “solar array$” OR “solar photovoltaic” OR “photovoltaic solar” OR “solar development$” OR “solar park$” OR “solar installation$” OR “solar facilit*” OR “solar plant$” OR “utility-scale solar energ*” OR biosolar OR “float* photovoltaic$” OR “float* solar” OR floatovoltaic$) AND (biodiversity OR ecolog* OR ecosystem$ OR *habitat$ OR species OR flor* OR vegetation$ OR animal$ OR faun* OR vertebrate$ OR mammal$ OR bird$ OR reptile$ OR amphibian$ OR invertebrate$ OR insect$ OR arthropod$ OR pollinator$ OR microbi* OR bacteri* OR microorganism$ OR fung* OR mushroom$)) | 1,428 | NA | Last accepted search  2 NOT 3 : 2600 hits = many relevant hits |
|  | 4 | ((photovoltaic$ OR "solar panel$" OR “solar array$” OR “solar development$” OR “solar power” OR “solar park$” OR “solar installation$” OR “solar facilit*” OR “solar plant$” OR “utility-scale solar energ*” OR “utility scale solar energ*” OR “solar renewable energ*” OR biosolar OR “float* solar” OR floatovoltaic$) AND (biodiversity OR ecolog* OR ecosystem$ OR *habitat$ OR species OR flor* OR vegetation$ OR animal$ OR faun* OR vertebrate$ OR mammal$ OR bird$ OR reptile$ OR amphibian$ OR invertebrate$ OR insect$ OR arthropod$ OR pollinator$ OR microbi* OR bacteri* OR microorganism$ OR fung* OR mushroom$)) | 4,085 | **NA** | Irrelevant hits  Florence, Florida instead flora  Not relevant “solar renewable energy” |
|  | 5 | ((photovoltaic$ OR "solar panel$" OR “solar array$” OR “solar development$” OR “solar power” OR “solar park$” OR “solar installation$” OR “solar facilit*” OR “solar plant$” OR “utility-scale solar energ*” OR “utility scale solar energ*” OR biosolar OR “float* solar” OR floatovoltaic$) AND (biodiversity OR ecolog* OR ecosystem$ OR *habitat$ OR species OR flora OR vegetation$ OR animal$ OR fauna OR vertebrate$ OR mammal$ OR bird$ OR reptile$ OR amphibian$ OR invertebrate$ OR insect$ OR arthropod$ OR pollinator$ OR microbi* OR bacteri* OR microorganism$ OR fung* OR mushroom$)) | 3,822 | 100% | Fauna and flora |
|  | 6 | ((photovoltaic$ OR "solar panel$" OR “solar array$” OR “solar development$” OR “solar power” OR “solar park$” OR “solar installation$” OR “solar facilit*” OR “solar plant$” OR “utility-scale solar energ*” OR “utility scale solar energ*” OR biosolar OR “float* solar” OR floatovoltaic$) AND (biodiversity OR ecolog* OR ecosystem$ OR *habitat$ OR species OR vegetation$ OR animal$ OR vertebrate$ OR mammal$ OR bird$ OR reptile$ OR amphibian$ OR invertebrate$ OR insect$ OR arthropod$ OR pollinator$ OR microbi* OR bacteri* OR microorganism$ OR fung* OR mushroom$)) | 3,810 | 100 % | Loss of some hits |
| **16/06/2022** | 1 | ((photovoltaic$ OR "solar panel$" OR “solar array$” OR “solar development$” OR “solar power” OR “solar park$” OR “solar installation$” OR “solar facilit*” OR “solar plant$” OR “utility-scale solar energ*” OR “utility scale solar energ*” OR biosolar OR “float* solar” OR floatovoltaic$) AND (biodiversity OR ecolog* OR ecosystem$ OR “natural habitat$” OR species OR flora OR vegetation$ OR animal$ OR fauna OR vertebrate$ OR mammal$ OR bird$ OR reptile$ OR amphibian$ OR invertebrate$ OR insect$ OR arthropod$ OR pollinator$ OR microbi* OR bacteri* OR microorganism$ OR fung* OR mushroom$)) | 3,792 | NA | Natural habitat in place of *habitat$ : some irrelevant references |
|  | 2 | ((photovoltaic$ OR "solar panel$" OR “solar array$” OR “solar development$” OR “solar power” OR “solar park$” OR “solar installation$” OR “solar facilit*” OR “solar plant$” OR “utility-scale solar energ*” OR “utility scale solar energ*” OR biosolar OR “float* solar” OR floatovoltaic$) AND (biodiversity OR ecolog* OR ecosystem$ OR “natural habitat$” OR species OR flora OR vegetation$ OR animal$ OR fauna OR vertebrate$ OR mammal$ OR bird$ OR reptile$ OR amphibian$ OR invertebrate$ OR insect$ OR arthropod$ OR microbi* OR bacteri* OR microorganism$ OR fung* OR mushroom$)) | 3,789 | NA | No pollinator because not homogeneous (too precise compared to other search terms) |
|  | 3 | ((photovoltaic$ OR "solar panel$" OR “solar array$” OR “solar development$” OR “solar power” OR “solar park$” OR “solar installation$” OR “solar facilit*” OR “solar plant$” OR “utility-scale solar energ*” OR “utility scale solar energ*” OR biosolar OR “float* solar” OR floatovoltaic$) AND (biodiversity OR ecolog* OR ecosystem$ OR “natural habitat$” OR species OR flora OR vegetation$ OR animal$ OR fauna OR vertebrate$ OR mammal$ OR bird$ OR reptile$ OR amphibian$ OR invertebrate$ OR insect$ OR arthropod$ OR microbi* OR bacteri* OR microorganism$ OR fung*)) | 3,770 | NA | No mushroom because not homogeneous |
|  | **4** | **((photovoltaic$ OR “solar panel$” OR “solar array$” OR “solar development$” OR “solar power” OR “solar park$” OR “solar installation$” OR “solar facilit*” OR “solar plant$” OR “utility-scale solar energ*” OR “utility scale solar energ*” OR biosolar OR “float* solar” OR floatovoltaic$) AND (biodiversity OR ecolog* OR ecosystem$ OR wildlife OR “natural habitat$” OR species OR flora OR vegetation$ OR animal$ OR fauna OR vertebrate$ OR mammal$ OR bird$ OR reptile$ OR amphibian$ OR invertebrate$ OR arthropod$ OR insect$ OR arachnid$ OR crustacean$ OR mollus* OR microbi* OR bacteri* OR microorganism$ OR fung*))** | 3,794 | 100 % | Arachnid, crustacean, molluscs  **=ACCEPTED FIANL SEARCH STRING** |
|  | 5 | ((photovoltaic$ OR "solar panel$" OR “solar array$” OR “solar development$” OR “solar power” OR “solar park$” OR “solar installation$” OR “solar facilit*” OR “solar plant$” OR “utility-scale solar energ*” OR “utility scale solar energ*” OR biosolar OR “float* solar” OR floatovoltaic$) AND (biodiversity OR ecolog* OR ecosystem$ OR wildlife OR “natural habitat$” OR species OR flora OR vegetation$ OR fern$ OR moss* OR animal$ OR fauna OR vertebrate$ OR mammal$ OR bat$ OR ungulate$ OR deer$ OR rodent$ OR felid$ OR cervid$ OR mustlid$ OR bird$ OR owl$ OR raptor$ OR passerine$ OR avian OR reptile$ OR snake$ OR turtle$ OR turtoise$ OR amphibian$ OR toad$ OR frog$ OR invertebrate$ OR arthropod$ OR insect$ OR ant$ OR butterfly* OR bee$ OR cricket$ OR grasshopper$ OR arachnid$ OR spider$ OR crustacean$ OR mollus* OR shellfish* OR microbi* OR bacteri* OR microorganism$ OR fung*)) | 51,605 | NA | Biodiversity terms amplified: ‘bee$’ can be ‘been’ |
|  | 6 | ((photovoltaic$ OR "solar panel$" OR “solar array$” OR “solar development$” OR “solar power” OR “solar park$” OR “solar installation$” OR “solar facilit*” OR “solar plant$” OR “utility-scale solar energ*” OR “utility scale solar energ*” OR biosolar OR “float* solar” OR floatovoltaic$) AND (biodiversity OR ecolog* OR ecosystem$ OR wildlife OR “natural habitat$” OR species OR flora OR vegetation$ OR fern$ OR moss* OR animal$ OR fauna OR vertebrate$ OR mammal$ OR bat$ OR ungulate$ OR deer$ OR rodent$ OR felid$ OR cervid$ OR mustlid$ OR bird$ OR owl$ OR raptor$ OR passerine$ OR avian OR reptile$ OR snake$ OR turtle$ OR turtoise$ OR amphibian$ OR toad$ OR frog$ OR invertebrate$ OR arthropod$ OR insect$ OR ant$ OR butterfly* OR cricket$ OR grasshopper$ OR arachnid$ OR spider$ OR crustacean$ OR mollus* OR shellfish* OR microbi* OR bacteri* OR microorganism$ OR fung*)) | 5,861 | NA | Not bee$ |
|  | 7 | ((photovoltaic$ OR "solar panel$" OR “solar array$” OR “solar development$” OR “solar power” OR “solar park$” OR “solar installation$” OR “solar facilit*” OR “solar plant$” OR “utility-scale solar energ*” OR “utility scale solar energ*” OR biosolar OR “float* solar” OR floatovoltaic$) AND (biodiversity OR ecolog* OR ecosystem$ OR wildlife OR “natural habitat$” OR species OR flora OR vegetation$ OR fern$ OR moss OR mosses OR animal$ OR fauna OR vertebrate$ OR mammal$ OR bat$ OR ungulate$ OR deer$ OR rodent$ OR felid$ OR cervid$ OR mustlid$ OR bird$ OR owl$ OR raptor$ OR passerine$ OR avian OR reptile$ OR snake$ OR turtle$ OR turtoise$ OR amphibian$ OR toad$ OR frog$ OR invertebrate$ OR arthropod$ OR insect$ OR ant$ OR butterfly* OR bee OR bees OR cricket$ OR grasshopper$ OR arachnid$ OR spider$ OR crustacean$ OR mollus* OR shellfish* OR microbi* OR bacteri* OR microorganism$ OR fung*)) | WOSCC: 7,631 | NA | Doubles corpus size for WOSCC = not feasible for our map |

# **Search string for Scopus**

**Test list search string** (– adapted to scopus format)

DOI(10.1088/1748-9326/11/7/074016) OR DOI(10.1007/s10841-019-00191-5) OR DOI(10.1016/j.vetpar.2012.04.016) OR DOI(10.46534/jliv.2017.04.03.009) OR DOI(10.1016/j.landurbplan.2013.11.017) OR DOI(10.1038/srep29235) OR DOI(10.1038/s41598-021-86756-4) OR DOI(10.1002/ep.12626) OR DOI(10.3390/plants9091125) OR DOI(10.1111/j.1523-1739.2010.01518.x) OR DOI(10.1016/j.jhydrol.2018.11.019) OR DOI(10.1371/journal.pone.0232034) OR DOI(10.1002/ldr.3408) OR DOI(10.3389/fmicb.2018.03043) OR DOI(10.1016/j.jenvman.2018.08.017) OR DOI(10.1007/s00267-017-0906-4) OR DOI(10.1007/s10841-016-9897-3) OR DOI(10.1002/ecs2.3089) OR DOI(10.1002/eap.2349) OR DOI(10.1111/1758-2229.12608) OR DOI(10.1111/1751-7915.13620) OR DOI(10.1016/j.renene.2018.08.106) OR DOI(10.1016/j.renene.2016.02.041) OR DOI(10.1371/journal.pone.0243296) OR DOI(10.1080/15659801.2015.1045791) OR TITLE(“REVEGETATION IN SOLAR PHOTOVOLTAIC FARMS IN MEDITERRANEAN AREAS”)

=> On the 26, 25 were found on Scopus on 3 June 2022

| **Date** | **Search string** | **Hits** | **Comprehensiveness** | **Comments** |
| --- | --- | --- | --- | --- |
| 03/06/2022 | (("photovoltaic panel" OR "photovoltaic cell" OR “photovoltaic array” OR “photovoltaic power” OR "solar panel" OR “solar array” OR “solar photovoltaic” OR “photovoltaic solar” OR “solar development” OR “solar park” OR “solar installation” OR “solar facilit*” OR “solar plant” OR “utility-scale solar energ*” OR biosolar OR “float* photovoltaic” OR “float* solar” OR floatovoltaics) AND (biodiversity OR ecolog* OR ecosystem OR *habitat OR species OR flor* OR vegetation OR animal OR faun* OR vertebrate OR mammal OR bird OR reptile OR amphibian OR invertebrate OR insect OR arthropod OR pollinator OR microbi* OR bacteri* OR microorganism OR fung* OR mushroom)) | Scopus : 2,661 on TITLE-ABS-KEY | Scopus: 25/25 = 100 % | NA |
| 10/06/2022 | ((photovoltaic OR "solar panel" OR “solar array” OR “solar development” OR “solar power” OR “solar park” OR “solar installation” OR “solar facilit*” OR “solar plant” OR “utility-scale solar energ*” OR “utility scale solar energ*” OR biosolar OR “float* solar” OR floatovoltaic) AND (biodiversity OR ecolog* OR ecosystem OR *habitat OR species OR flora OR vegetation OR animal OR fauna OR vertebrate OR mammal OR bird OR reptile OR amphibian OR invertebrate OR insect OR arthropod OR pollinator OR microbi* OR bacteri* OR microorganism OR fung* OR mushroom)) | Scopus : 6,222 on TITLE-ABS-KEY | Scopus: 25/25 = 100 % | NA |
| 16/06/2022 | ((photovoltaic OR “solar panel” OR “solar array” OR “solar development” OR “solar power” OR “solar park” OR “solar installation” OR “solar facilit*” OR “solar plant” OR “utility-scale solar energ*” OR “utility scale solar energ*” OR biosolar OR “float* solar” OR floatovoltaic) AND (biodiversity OR ecolog* OR ecosystem OR wildlife OR “natural habitat” OR species OR flora OR vegetation OR animal OR fauna OR vertebrate OR mammal OR bird OR reptile OR amphibian OR invertebrate OR arthropod OR insect OR arachnid OR crustacean OR mollus* OR microbi* OR bacteri* OR microorganism OR fung*)) | Scopus : 6,128 on TITLE-ABS-KEY | Scopus: 25/25 = 100 % | NA |

# **Search string for Google Scholar**

| Date | Search string number | Search string | Hits | Status |
| --- | --- | --- | --- | --- |
| 28/06/2022 | 1 | biodiversity AND (photovoltaic OR solar OR floatovoltaic OR floatovoltaics) | 59 | Retained |
| 28/06/2022 | 2 | vegetation AND (photovoltaic OR solar OR floatovoltaic OR floatovoltaics) | 348 | Retained |
| 28/06/2022 | 3 | (animal OR animals) AND (photovoltaic OR solar OR floatovoltaic OR floatovoltaics) | 284 | Retained |
| 28/06/2022 | 4 | (microorganism OR microorganisms) AND (photovoltaic OR solar OR floatovoltaic OR floatovoltaics) | 118 | Retained |
| 28/06/2022 | 5 | (vertebrates OR invertebrates) AND (photovoltaic OR solar OR floatovoltaic OR floatovoltaics) | 4 hits (1 article) | Not retained |
